# Cancer stem cells and post-therapy tumour recurrence: a systematic review of mechanistic pathways and translational gaps

**DOI:** 10.3332/ecancer.2025.2016

**Published:** 2025-10-16

**Authors:** Imad Barjij, Meryem Meliani

**Affiliations:** 1 Medical Oncology Department, National Institute of Oncology, Ibn Sina University Hospital, Rabat 10000, Morocco; 2 Faculty of Medicine and Pharmacy of Rabat, Mohammed V University of Rabat, Rabat 10000, Morocco; 3 Medical Oncology Department, Regional Oncology Center of Laâyoune, Regional Hospital Center of Laâyoune, Laâyoune 70000, Morocco; a https://orcid.org/0009-0004-2172-1058

**Keywords:** cancer stem cells, recurrence, therapy resistance, tumour relapse, epigenetics, EMT, tumour microenvironment, immunoevasion, mechanistic pathways, systematic review

## Abstract

**Background:**

Cancer stem cells (CSCs) are increasingly recognised as pivotal drivers of tumour recurrence and treatment resistance across multiple malignancies. Despite extensive preclinical investigations, the mechanisms by which CSCs mediate relapse after therapy remain insufficiently integrated and poorly translated into clinical frameworks.

**Objective:**

This systematic review aimed to synthesise current mechanistic evidence linking CSC biology to post-therapeutic recurrence in solid and hematologic tumours, highlighting recurrent molecular pathways, experimental models and translational gaps.

**Methods:**

Following Preferred Reporting Items for Systematic Reviews and Meta-Analyses 2020 guidelines, five major databases (PubMed, Scopus, Web of Science, Embase and ResearchRabbit) were searched without time or language restriction. Eligible studies included original experimental articles investigating the mechanistic role of CSCs in tumour recurrence, therapy resistance or metastatic relapse. A total of 23 studies were included after rigorous screening and data extraction. Risk of bias was assessed using predefined methodological criteria. No meta-analysis was conducted due to mechanistic and qualitative heterogeneity.

**Results:**

Included studies spanned diverse tumour types, including glioblastoma, breast, pancreatic, hepatocellular, colorectal, lung, thyroid and hematologic cancers. CSC-related recurrence was linked to key mechanistic axes: chromatin remodeling (e.g., suppressor of variegation 3-9 Homolog 1, methyltransferase like 16), transcriptional regulators (e.g., SRY-box transcription factor 2, MYC and Transcription factor activating enhancer-binding protein 4), epithelial-to-mesenchymal transition-associated plasticity, immune evasion (Programmed death-ligand 1, stimulator of interferon genes pathway suppression), metabolic rewiring (P-element induced WImpy Testis-like 2–PDK1, ribosomal biosynthesis) and microenvironmental crosstalk (cancer-associated fibroblasts- and myeloid-derived suppressor cells-mediated niches). Across studies, CSCs demonstrated higher resistance to chemotherapy, prolonged survival under treatment stress and robust capacity for tumour regeneration.

**Limitations:**

The majority of studies were preclinical and varied in CSC definitions and recurrence models. Few incorporated longitudinal tracking or patient-level validation. Overall risk of bias was moderate due to lack of blinding, protocol registration or replication.

**Conclusion:**

CSC-driven recurrence is a multifaceted and dynamic process shaped by epigenetic, transcriptional, metabolic and immunologic adaptations. Single-target strategies are unlikely to achieve durable eradication. Future research must prioritise multi-targeted approaches, integrate CSC endpoints into clinical trials and develop predictive biomarkers of CSC burden. Addressing CSC-mediated relapse is essential for advancing precision oncology and achieving lasting therapeutic responses.

## Introduction

### Background and rationale

Tumour recurrence following initial response to therapy remains one of the most critical challenges in oncology today. Despite significant advances in targeted therapies, immunotherapy and precision medicine, a substantial proportion of patients experience relapse, often more aggressive and treatment-resistant than the primary disease. Over the past two decades, a growing body of evidence has attributed this phenomenon, at least in part, to the persistence and adaptability of cancer stem cells (CSCs), a subpopulation of tumour cells endowed with self-renewal capacity, multipotency and resistance to conventional cytotoxic agents [1–7].

CSCs have now been identified in a wide spectrum of malignancies, including glioblastoma, breast cancer, hepatocellular carcinoma (HCC), colorectal cancer, non-small cell lung cancer (NSCLC), cervical cancer, ovarian carcinoma, pancreatic adenocarcinoma and hematologic cancers such as acute myeloid leukaemia (AML). These cells are not only capable of initiating tumours but are also implicated in key processes that drive therapeutic resistance, tumour dormancy, epithelial-to-mesenchymal transition (EMT), immune escape, metabolic reprogramming and eventual relapse. Functionally, CSCs are characterised by specific surface markers (e.g., cluster of differentiation (CD)44, CD133, Aldehyde Dehydrogenase (ALDH1)), enhanced DNA repair capacity, epigenetic plasticity and a dynamic interaction with the tumour microenvironment (TME), including immune cells and stromal components [1, 5, 8–11].

While the conceptual framework of CSCs has been extensively explored, the precise mechanisms by which these cells evade treatment and regenerate tumours remain incompletely understood. Existing reviews have provided valuable insights into selected molecular pathways, such as Wnt/β-catenin, Hedgehog, Notch and PI3K/AKT, but often focus on individual tumour types or therapeutic classes. Few have attempted to systematically integrate experimental evidence across multiple cancer types, encompassing diverse mechanistic insights into how CSCs contribute to post-treatment recurrence [1, 2, 5, 7, 12].

Furthermore, the translational potential of CSC-targeting strategies is still limited by a lack of mechanistic clarity. Despite promising preclinical studies, efforts to eliminate CSCs or prevent their reactivation post-therapy have yet to yield broadly effective clinical applications. Contributing to this gap is the fragmented nature of existing data, often scattered across isolated *in vitro* studies or small-scale *in vivo* models, without comprehensive synthesis [5, 10, 13–15].

Given this context, there is a clear need for a structured and critical appraisal of original experimental research exploring the role of CSCs in post-therapy tumour recurrence. Such a synthesis is essential not only to identify conserved molecular themes and vulnerabilities but also to highlight evidence gaps and inform the rational development of CSC-directed therapeutic interventions. This is particularly relevant as emerging combination strategies, such as immunotherapy paired with CSC-targeting agents, gain traction in translational oncology [4, 6, 8, 10, 13, 16].

This review, therefore, aims to address this unmet need by systematically evaluating and integrating experimental studies that investigate CSCs in the context of therapeutic relapse, resistance and disease progression. Unlike narrative reviews or meta-analyses limited to clinical outcomes, this work focuses specifically on mechanistic pathways and experimental evidence, spanning *in vitro* assays, *in vivo* models (including patient-derived xenografts (PDXs)), organoid systems and omics-based approaches. In doing so, it provides a detailed landscape of CSC-driven recurrence mechanisms, identifies common regulatory networks and proposes translational directions grounded in experimental biology.

### Objectives

This systematic review aims to critically synthesise experimental evidence on the role of CSCs in post-therapy tumour recurrence, with an emphasis on mechanistic pathways and biological processes implicated in relapse, resistance and disease progression. Specifically, the review addresses the following objectives:

1. To identify and summarise original experimental studies (in vitro, in vivo, ex vivo or multi-omics) that explore the contribution of CSCs to tumour recurrence following treatment.
2. To classify and analyse the underlying molecular and cellular mechanisms enabling CSC survival, maintenance and expansion after therapy.
3. To highlight key evidence gaps and limitations in the current mechanistic literature and provide recommendations for future translational or therapeutic strategies.

This review follows the Preferred Reporting Items for Systematic Reviews and Meta-Analyses (PRISMA) 2020 reporting standards and is based on a rigorously curated set of 23 original studies (Table 1), selected according to strict inclusion criteria focused on CSC identification, experimental validity and relevance to therapeutic recurrence. To the best of our knowledge, this is the first systematic synthesis that bridges experimental oncology and stem cell biology to comprehensively map CSC-driven relapse mechanisms across diverse tumour entities.

## Methods

### Eligibility criteria

We defined clear and stringent eligibility criteria to ensure the inclusion of original experimental studies with high mechanistic value related to CSCs and post-therapy tumour recurrence. Eligible studies had to meet the following criteria:

- Study type: Only original experimental studies were included. This encompassed *in vitro* studies, *in vivo* animal models (e.g., xenografts, syngeneic or transgenic models), organoid models, PDXs and multi-omics investigations. Studies combining these methods were prioritised.
- Cancer type: All solid tumours and hematologic malignancies were considered eligible if CSCs were explicitly investigated in the context of therapeutic relapse or recurrence.
- CSC focus: Studies must have clearly identified CSCs or tumour-initiating cells (TICs) using established markers (e.g., CD44, CD133, ALDH1, extramedullary infiltration (EpCAM)) or functional assays (e.g., tumoursphere formation and extreme limiting dilution assay (ELDA)). Studies lacking specific CSC markers or mechanistic data were excluded.
- Outcome of interest: Only studies assessing recurrence, resistance or survival advantage of CSCs post-therapy were included. Studies focused solely on tumourigenesis without any link to therapy or relapse were excluded.
- Publication characteristics: Only full-text, peer-reviewed articles in English were included. Reviews, editorials, commentaries, conference abstracts and study protocols were excluded. Time frame: No date restriction was applied to the initial search. While the majority of eligible studies were published between 2003 and 2024, the review also included studies published as recently as 2025, enhancing the recency and relevance of the synthesis.

Included studies were grouped and synthesised according to cancer type and mechanistic pathway (e.g., Wnt/β-catenin, EMT and immune evasion), as reflected in Table 1.

### Information sources

To identify relevant studies, we searched the following bibliographic databases:

- PubMed/MEDLINE (via NCBI)
- Scopus (Elsevier)
- Web of Science Core Collection (Clarivate Analytics)
- Embase (Elsevier)
- Google Scholar (manual selection from first 200 hits)

All databases were searched between 15 January and 10 February 2025.

Additionally, we manually screened the reference lists of included articles and relevant systematic reviews to identify studies not captured through database searches.

No grey literature, trial registries, unpublished data or non-English articles were included.

### Search strategy

Search strategies combined MeSH terms and free-text keywords. An example of the PubMed strategy is as follows:

(‘CSC’ OR ‘TIC’ OR ‘TIC’ OR ‘CSCs’ OR ‘TICs’) AND (‘recurrence’ OR ‘relapse’ OR ‘resistance’ OR ‘residual disease’ OR ‘post-therapy’) AND (‘mechanism’ OR ‘pathway’ OR ‘signaling’ OR ‘molecular’) AND (‘*in vitro*’ OR ‘*in vivo*’ OR ‘xenograft’ OR ‘PDX’ OR ‘organoid’ OR ‘transcriptomic’ OR ‘multi-omics’).

We applied filters for English language and peer-reviewed journal articles. No restriction on publication date was imposed.

### Selection process

Two reviewers (I.

B. and M.M.) independently screened titles and abstracts. Full texts were retrieved for all studies deemed potentially eligible. Disagreements were resolved through discussion between the two reviewers.

**Figure 1.**
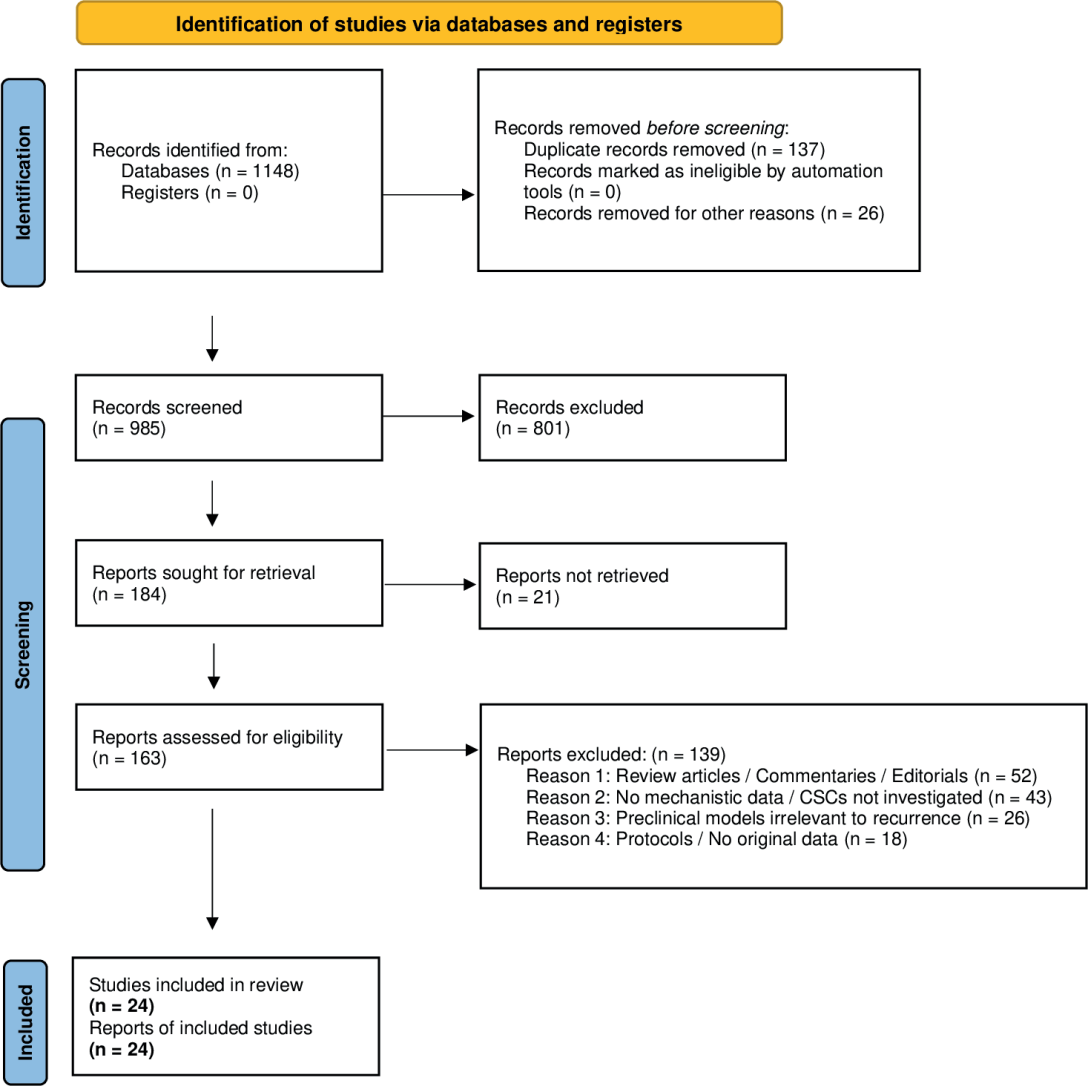
PRISMA flow diagram.

The study selection process is illustrated in Figure 1 below. Of the 1,148 records initially identified, 985 were screened after removal of duplicates and non-eligible records. Following full-text assessment of 163 reports, a total of 23 studies met the predefined inclusion criteria and were incorporated into the final qualitative synthesis.

### Data collection process

Two reviewers independently extracted data from each included study using a pre-designed template. The data collection focused on study design, model/system used, cancer type, CSC identification method, treatment applied, type of recurrence investigated, molecular mechanisms described and key outcomes. Any disagreements were resolved through consensus.

No automation tools were used. When information was missing or unclear, supplementary materials or full-text reanalysis were consulted. No authors were contacted.

### Data items

#### Outcomes

The primary outcome was the mechanistic role of CSCs in post-therapy tumour recurrence, including: - Tumour regrowth or resistance after chemotherapy, radiotherapy or targeted therapy - CSC enrichment or maintenance following treatment - Identification of signaling pathways or epigenetic changes driving CSC-mediated relapse.

Secondary outcomes included: - Immune evasion mechanisms - Metastatic dormancy and late relapse - Therapeutic response modulation via CSC pathways.

#### Other variables

Other variables extracted included:

- Cancer type and subtype
- Model system (cell lines, organoids and animal models)
- Type and duration of treatment applied
- Functional assays used to define CSC activity
- Omics techniques (e.g., RNA sequencing (RNA-seq), ATAC-seq and chromatin immunoprecipitation sequencing (ChIP-seq))

When data were missing (e.g., duration of follow-up or expression levels), we documented the lack transparently without imputation.

### Risk of bias assessment

Risk of bias refers to the potential for systematic errors that could distort the interpretation of a study’s findings. In this review, the risk of bias was assessed for each study by two reviewers based on methodological rigor:

- Type of model used (e.g., PDX versus cell line)
- Clarity and reproducibility of CSC identification
- Presence of functional validation (e.g., limiting dilution assays)
- Relevance to recurrence or resistance

No standardised risk-of-bias tool exists for mechanistic CSC studies; we therefore developed a qualitative judgment scale with three levels: Low, moderate and high, justified in Table 1. Disagreements in assessment were resolved by consensus.

### Effect measures

No quantitative synthesis or meta-analysis was planned, as this review focused on mechanistic pathways rather than effect sizes. As such, no statistical effect measures (e.g., odds ratios and hazard ratios) were applied. A meta-analysis was deemed methodologically inappropriate due to the heterogeneity of experimental designs, model systems, outcome definitions and mechanistic endpoints across the included studies.

Instead, outcomes were reported narratively and synthesised through thematic classification of biological mechanisms (e.g., Wnt activation and metabolic reprogramming).

### Synthesis methods

We tabulated study characteristics and outcomes in Table 1. Studies were grouped according to:

- Cancer type (e.g., glioblastoma, breast cancer and AML)
- Experimental system (e.g., *in vitro*, *in vivo* and multi-omics)
- Pathway or mechanism (e.g., EMT, immune escape and chromatin regulation)

This classification enabled a structured narrative synthesis highlighting converging mechanisms and evidence gaps.

We did not conduct sensitivity analyses or subgroup stratifications due to the descriptive nature of the synthesis.

### Reporting bias and certainty assessment

We did not conduct formal assessments of publication bias or certainty of evidence (e.g., GRADE), as this review is not focused on intervention outcomes or effect estimation.

However, we qualitatively discuss potential gaps in the literature, including overrepresentation of certain cancer types and underreporting of immune-based CSC mechanisms.

## Results

### Study selection

A total of 23 original studies were selected based on the predefined eligibility criteria. Figure 1 provides a detailed summary of the selection process in accordance with PRISMA 2020 guidelines.z

### Characteristics of included studies

The 23 included studies span publication years from 1997 to 2025, with a sharp increase in publications from 2022 onward and a notable concentration of high-quality mechanistic studies published in 2024 and 2025. The included articles cover a broad spectrum of solid and hematologic malignancies, including glioblastoma, pancreatic ductal adenocarcinoma, AML, breast cancer, HCC, NSCLC, colorectal cancer, triple-negative breast cancer (TNBC), ovarian cancer and chordoma.

Most studies used multi-platform designs, integrating *in vitro*, *in vivo* and omics approaches. CSCs were identified using canonical markers (e.g., CD44, CD133 and ALDH1), functional assays (e.g., tumoursphere formation, label-retention and ELDA) and transcriptomic signatures. Several studies employed xenograft or patient-derived models (PDX), while others incorporated clinical correlation data, notably those by Ginestier *et al* [17], Song *et al* [18] and Yang *et al* [19].

### Risk of bias

Risk of bias was assessed qualitatively based on study design, mechanistic rigor, translational relevance and completeness of outcome reporting. The majority of studies were classified as having moderate risk of bias, primarily due to lack of longitudinal patient follow-up, absence of blinded outcome assessments or reliance on single model systems. Only three studies were rated as low risk, all of which included multi-level validation, large patient cohorts or integration of functional assays with clinical data. A justification for each risk rating is provided in Table 1, alongside key mechanistic findings.

### Summary of key mechanistic themes driving post-therapy CSC persistence and recurrence

Several recurrent mechanisms and biological themes emerged from the 23 included studies:

- Chromatin remodeling and epigenetic plasticity: Li *et al* [21] demonstrated that chromatin regulators such as suppressor of variegation 3-9 Homolog 1 (SUV39H1) and NR5A2 maintain CSC stemness and mediate therapy resistance through altered enhancer landscapes and transcriptional plasticity.
- Non-canonical signaling and immune evasion: TME-driven pathways, such as Wnt5a–CREB1–BACH1, programmed death-ligand 1 (PD-L1) upregulation and stimulator of interferon genes (STING) pathway suppression, e.g., [2, 22, 23], were shown to enhance CSC survival post-therapy by impairing immune surveillance and promoting niche-mediated dormancy.
- Metabolic reprogramming: Several studies linked CSC survival to enhanced glycolysis, mitochondrial adaptation and ribosome biogenesis, e.g., [24, 25], often driven by upstream regulators such as P-element induced WImpy Testis-like 2 (PIWIL2), Mammalian target of rapamycin (mTOR) and Methyltransferase Like 16 (METTL16).
- CSC plasticity and reprogramming: Studies such as Peyvandi *et al* [33] and Sarkar *et al* [37] highlighted CSC interconversion and plasticity, particularly in response to inflammatory or chemotherapeutic cues, emphasising that CSCs may re-emerge from non-stem-like populations under stress.
- Apoptotic and differentiation escape: Modulators of apoptosis (e.g., *miR-34*, *Notch* and *Bcl-2*) and epigenetic repressors (*HDAC* and *EZH2*) were recurrently implicated in preventing therapy-induced cell death or promoting incomplete differentiation.

### Heterogeneity across cancer types

The included studies reflect considerable heterogeneity in terms of cancer type, CSC identification method and experimental system. While some mechanisms, such as Wnt signaling, ALDH1-associated resistance and immune escape ligands, were consistently reported across several cancers, others appeared tumour-specific, such as NR5A2-driven recurrence in PDAC or CD73-associated immune evasion in TNBC.

To facilitate comparison, full study characteristics and extracted data are presented in Table 1.

### Excluded studies

Among the 139 full-text articles excluded, the majority were non-original reports such as reviews, editorials or commentaries (*n* = 52). Forty-three studies lacked investigation of cancer stem cells or failed to provide mechanistic data related to recurrence. Twenty-six reports explored preclinical models unrelated to relapse or resistance, and 18 articles were excluded for being protocols or reports without experimental data. Excluded studies were documented and archived for transparency.

### Certainty of evidence

While the majority of included studies demonstrate high internal validity and mechanistic plausibility, the certainty of evidence remains moderate overall, owing to limited patient-level outcome data, absence of clinical trials and heterogeneous CSC markers. Studies integrating functional validation, PDXs and patient correlation, e.g., [17, 18, 36] contributed most to the certainty assessment.

## Discussion

### General interpretation of findings

This systematic review synthesised mechanistic evidence from 23 original studies exploring the role of CSCs in mediating tumour recurrence across a wide spectrum of solid and hematologic malignancies. Despite significant heterogeneity in study designs, cancer types and experimental models, several overarching mechanistic themes emerged. These included transcriptional plasticity, chromatin remodeling, epigenetic regulation, immunoevasion, metabolic reprogramming and microenvironmental interactions. Collectively, the data converge toward a unified concept: CSCs persist through and beyond therapy, driving relapse through dynamic adaptation mechanisms and evasion of cytotoxic pressures [1, 3, 5–7, 38].

Across multiple tumour models (glioblastoma, breast, pancreatic, colorectal, hepatocellular, lung, cervical and thyroid carcinomas), studies consistently reported that CSC-enriched subpopulations demonstrate higher resistance to conventional therapies, enhanced self-renewal capacity and increased metastatic potential. This is exemplified by studies such as Zheng *et al* [36], where inhibition of specific chromatin regulators (SUV39H1 and NR5A2, respectively) restored chemosensitivity in resistant CSC compartments. Similarly, Gupta *et al* [29] and Mediratta *et al* [32] showed that targeting EMT-related pathways or metabolic axes significantly abrogated CSC-driven tumour regrowth and metastasis [9, 10, 14, 20, 21, 33, 37, 39, 40].

These results underscore a critical therapeutic paradox: while bulk tumour cells may respond to treatment, CSCs survive and re-initiate tumours through latent and resistant states. Dormancy and reactivation mechanisms, as illustrated by Bushnell *et al* [23], suggest a temporally regulated escape from immune surveillance mediated by reduced STING signaling and transcriptional rewiring. Other studies implicated the TME in CSC maintenance, such as Fang *et al* [22] and Peyvandi *et al* [33], demonstrating that cancer-associated fibroblasts (CAFs) and myeloid-derived suppressor cells create a niche that supports CSC survival and plasticity [4, 9, 10, 23, 33, 41].

### Conceptual synthesis: major mechanistic pathways

**Figure 2.**
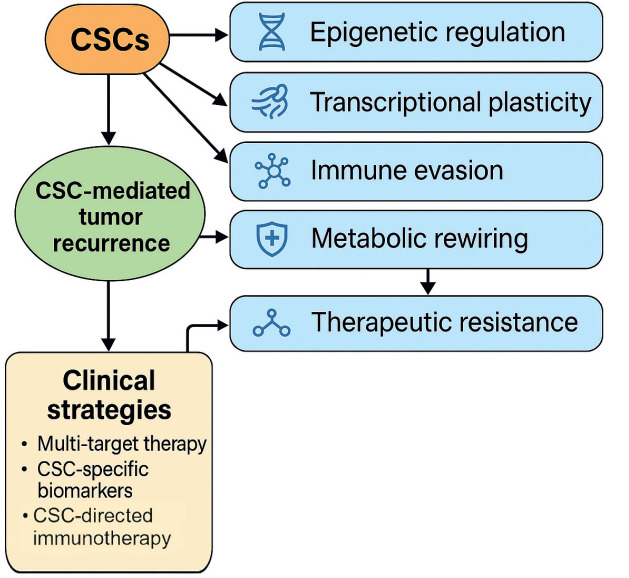
Conceptual diagram of CSC-mediated recurrence mechanisms and biological complexity. Conceptual representation of the major biological mechanisms contributing to CSC-mediated recurrence. The diagram illustrates how epigenetic regulation, transcriptional plasticity, immune evasion, metabolic rewiring and TME-driven reinforcement interact to sustain CSC persistence and therapeutic resistance. The diversity and interplay of these mechanisms underscore the intrinsic biological complexity of CSC-mediated relapse.

Based on the aggregated evidence, we propose a conceptual framework integrating the main pathways underlying CSC-mediated recurrence (Figure 2). These include: [2, 9, 10, 14, 15, 19, 24, 39, 42, 43]

- Epigenetic regulators (e.g., SUV39H1, METTL16) that maintain an open chromatin state for stemness gene expression.
- Transcriptional programs (e.g., SRY-box transcription factor 2 (SOX2), MYC, transcription factor activating enhancer-binding protein 4 (TFAP4)) that govern self-renewal and dedifferentiation.
- EMT and mesenchymal transition pathways that promote invasiveness and chemoresistance.
- Immune evasion mechanisms, including upregulation of PD-L1 and loss of immunogenicity.
- Metabolic rewiring, such as glycolysis activation (via PIWIL2–PDK1 axis) and ribosomal biogenesis.
- TME-driven reinforcement, particularly from CAFs and tumour-educated immune cells.

These pathways are not mutually exclusive; rather, they act in a networked and sometimes redundant fashion to ensure CSC survival. As shown by several studies [18, 21, 34], single-pathway inhibition rarely achieves durable eradication of CSCs, thus supporting the need for multi-targeted strategies [4, 14, 15].

### Limitations of the evidence base

Although mechanistically rich, the evidence included in this review is subject to several limitations. First, the majority of studies were preclinical, relying on cell lines, PDXs or mouse models. While these systems offer experimental tractability, they may not fully recapitulate human tumour heterogeneity or immune contexture. Only a minority of studies [17, 18] incorporated substantial patient cohort validation, which limits the generalisability of many findings [13, 17, 18, 44–48].

Second, definitions of CSCs varied considerably across studies. Some relied on phenotypic markers (e.g., CD44+/CD24– and ALDHhigh), others on functional assays (e.g., tumoursphere formation and label retention) and yet others used transcriptomic signatures. This lack of standardisation complicates direct comparison and synthesis. Moreover, recurrence itself was operationalised heterogeneously, ranging from *in vivo* tumour regrowth post-treatment, to resistance to chemotherapy, to expression of relapse-associated gene programs [9, 13, 15, 39, 49].

Third, few studies have conducted longitudinal assessments of CSC dynamics during and after therapy. Most findings were derived from endpoint analyses rather than temporal tracking. Consequently, we lack a detailed understanding of how CSC populations evolve under treatment pressure, an essential aspect for designing effective eradication strategies [13, 19, 44, 48, 50].

Lastly, the risk of bias in included studies was predominantly moderate, largely due to the absence of blinding, replication or standardised reporting of negative results. None of the studies employed registered protocols and reporting completeness varied.

### Limitations of the review process

This review has some intrinsic limitations despite rigorous adherence to PRISMA 2020 guidelines. First, although our search strategy was comprehensive (across five major databases), gray literature and unpublished studies were not included, possibly leading to publication bias. Second, screening, data extraction and bias assessment were conducted by two independent reviewers (I.

B. and M.M.), with any disagreements resolved through discussion. While this approach is robust, the absence of a third adjudicator may introduce subjectivity in borderline inclusion decisions [6, 15, 16, 41, 48, 51].

Additionally, no meta-analysis was performed, as the review focused on mechanistic evidence, which is inherently qualitative and heterogenous. Although this limits quantitative synthesis, the narrative integration provided a detailed and translationally meaningful overview of biological pathways [26, 33, 36, 41, 52].

Finally, while the review includes studies up to 2025, reflecting high contemporaneity, it remains a snapshot and does not incorporate living review updates.

### Implications for practice, policy and future research

This synthesis has several important implications:

- Therapeutic targeting: CSCs are clearly central to recurrence. However, monotherapies against individual pathways (e.g., Bcl-2 inhibition and Wnt blockade) are unlikely to suffice. Rational combinatorial therapies, targeting epigenetic regulators, metabolic pathways and immune checkpoints, should be prioritised [2, 4, 5, 10, 15].
- Biomarker development: Current CSC markers are heterogeneous and context-dependent. Multi-parametric approaches combining surface markers, transcriptomic profiles and functional assays will be essential to reliably identify CSCs and monitor treatment response [8, 10, 13, 15, 47].
- Clinical trial design: Many clinical trials fail to consider CSC dynamics. Future trials should include CSC burden as an endpoint and utilise longitudinal biopsies or liquid biopsies to assess CSC persistence [2, 10, 13, 47, 50].
- Immunotherapy: The role of CSCs in immune evasion (via PD-L1, natural killer (NK) ligand modulation and dormancy) suggests that CSC-targeted immunotherapies, either via CSC vaccines or NK-based approaches, could augment existing checkpoint inhibitors [4, 5, 8, 10, 23, 32, 33].
- Preclinical modeling: Investment in organotypic cultures, humanised mouse models and time-resolved single-cell approaches is critical to bridge the gap between discovery and clinical translation [45, 53, 54].
- Policy and funding: Given the high cost of late-stage relapse in cancer care, funding agencies should prioritise studies dissecting CSC-targeted strategies and support precision oncology platforms that incorporate stemness profiling [4–6].

In conclusion, this systematic review provides a comprehensive synthesis of 23 original studies elucidating the mechanistic roles of CSCs in cancer recurrence. CSC-mediated relapse involves a complex interplay of genetic, epigenetic, immune and microenvironmental factors. Future therapeutic and diagnostic advances must embrace this complexity and integrate CSC biology into mainstream oncologic paradigms.

Figure 2 below provides a conceptual visual summary of the major pathways involved in CSC-mediated recurrence and highlights the critical knowledge gaps that remain.

Although the development of CSC-targeted strategies has been extensively explored in preclinical settings, translation into clinical trials remains limited. A few early-phase clinical trials have attempted to target CSC-specific pathways or markers. For example, the use of demcizumab (anti-DLL4 monoclonal antibody) in pancreatic and NSCLC showed some initial promise [20], although subsequent trials were terminated due to toxicity and limited efficacy. Another example includes BBI608 (napabucasin), a STAT3 inhibitor investigated in several trials [55, 56], which aimed to target CSC-associated signaling. However, many of these efforts failed to demonstrate consistent clinical benefits. As of today, no randomised clinical trial has definitively validated a CSC-specific therapy as a standard of care. This underscores the persistent gap between mechanistic insights and therapeutic translation.

## Conclusion

CSCs are central drivers of tumour recurrence and therapeutic resistance across diverse cancer types. Despite heterogeneous models, recurrent mechanisms such as chromatin remodeling, immune evasion and metabolic plasticity consistently underlie CSC-mediated relapse. Effective cancer control must integrate CSC-targeted strategies, including immunotherapeutic approaches and biomarker-guided treatments. Recognising CSCs as pivotal entities in recurrence is essential for advancing toward durable remission and curative therapies.

## List of abbreviations

ALDH, Aldehyde dehydrogenase; AML, Acute myeloid leukaemia; ATC, Anaplastic thyroid cancer; CAF, Cancer-associated fibroblast; CD, Cluster of differentiation; ChIP-seq, Chromatin immunoprecipitation sequencing; CSC, Cancer stem cell; DDP, Cisplatin (Diamminedichloroplatinum); DIM, 3,3’-Diindolylmethane; DOX, Doxorubicin; e-DDMSNP, Exosome-coated dendritic mesoporous silica nanoparticle; ELDA, Extreme limiting dilution assay; EMI, Extramedullary infiltration; EMT, Epithelial-to-mesenchymal transition; EpCAM, Epithelial cell adhesion molecule; FACS, Fluorescence-activated cell sorting; GSC, Glioblastoma stem cell; HCC, Hepatocellular carcinoma; HDACi, Histone deacetylase inhibitor; IHC, Immunohistochemistry; KO, Knockout; METTL16, Methyltransferase like 16; mTOR, Mammalian target of rapamycin; NK, Natural killer (cell); NSCLC, Non-small cell lung cancer; PD-L1, Programmed death-Ligand 1; PDO, Patient-derived organoid; PDX, Patient-derived xenograft; PIWIL2, P-element induced WImpy Testis-like 2; PRISMA, Preferred Reporting Items for Systematic Reviews and Meta-Analyses; qRT-PCR, Quantitative real-time polymerase chain reaction; RNA-seq ,RNA sequencing; SCC, Squamous cell carcinoma; scRNA-seq, Single-cell RNA sequencing; SOX2, SRY-box transcription factor 2; SP, Side population; STING, Stimulator of interferon genes; SUV39H1, Suppressor of variegation 3-9 Homolog 1; TFAP4, Transcription factor activating enhancer-binding protein 4; TIC, Tumour-initiating cell; TMA, Tissue microarray; TME, Tumour microenvironment; TMZ, Temozolomide; ULBP1, UL16-Binding Protein 1; Wnt-C59, Porcupine inhibitor targeting Wnt signaling.

## Conflicts of interest

The authors declare no conflicts of interest related to the content of this systematic review. No financial or personal relationships influenced the design, analysis or interpretation of the results.

## Funding

This study received no specific grant from any funding agency in the public, commercial or not-for-profit sectors. It was conducted as part of the academic and scientific engagement of the authors.

## Ethical approval

As this work is a systematic review of published data, no ethical approval or informed consent was required. All included studies were already approved by their respective institutional review boards.

## Data availability

All data generated or analysed during this systematic review are either included in the main article. The complete data extraction table (Table 1), PRISMA flow diagram (Figure 1) and conceptual synthesis figure (Figure 2) are available within the manuscript. Additional information can be obtained from the corresponding author upon request.

## Figures and Tables

**Table 1.** Summary of included studies and key findings.

Reference (Author, Year) (ref)	Study type	Model / System	Cancer type	CSC identification method	Treatment applied (if any)	Type of recurrence investigated	Mechanisms involved	Key findings	Level of evidence	Risk of bias
Kimura *et al* (2024) [26]	*In vitro* + *In vivo* study	Human ATC cell lines (THJ-11T, THJ-16T, 8505C); xenografts in nude mice	Anaplastic Thyroid Cancer (ATC)	SORE6 fluorescent reporter, ALDH assay, tumorsphere formation	Dabrafenib, Trametinib, Combo BRAF+MEK	Post-treatment residual CSC activity	MEK inhibition suppresses CSC-related transcription, dampens Pol II activity	Trametinib (MEK inhibitor) strongly inhibits CSC activity; combined therapy reduces but does not eliminate CSCs; residual CSCs may explain recurrence post-therapy in ATC.	Preclinical, robust	Moderate (no long-term follow-up of residual CSC fate post-treatment)
Yang *et al* (2022) [19]	*In vitro* + *in vivo* + omics	Primary AML samples (*n* = 50); PDX in NSG mice; scRNA-seq; flow cytometry	AML	C1Q+ macrophage-like population, FCGR3A+, MAFB+ via scRNA-seq and FACS	Azacytidine, CAG, CLAG, Decitabine, FLAG (clinical context)	Extramedullary infiltration + early relapse	Complement signaling; fibroblast-mediated migration; TGF-β1 activation	C1Q+ leukemia cells represent a macrophage-like subclone with high migratory capacity; associated with EMI, relapse, and poor survival. Fibroblast-C1Q axis promotes migration and chemoresistance. Depletion of C1Q+ cells delays progression in mice.	Translational (multi-platform)	Moderate (strong multimodal evidence, but single-patient basis for key *in vivo* insights)
Bonnet *et al* (1997) [27]	*In vivo* + *ex vivo* functional	Primary AML cells; SCID mouse engraftment; serial transplantation	AML	CD34+CD38– sorting; serial engraftment into immuno deficient mice	None (functional assay only)	Not directly assessed (focus on leukemia propagation)	Hierarchical model of AML with CSC-like primitive CD34+CD38– cells	Demonstrated for the first time that AML is hierarchically organized, with rare CD34+CD38– cells capable of initiating leukemia in SCID mice. These cells represent functionally defined CSCs but recurrence or resistance not explicitly studied.	Foundational preclinical	Moderate (pioneering but lacks recurrence or therapy data, limited CSC markers by today’s standards)
Bushnell *et al* (2024) [23]	*In vivo* (syngeneic mice) + *in vitro* + RNA-seq	Murine breast cancer cell lines (D2.0R, PyMT, Met1); label-retaining CSCs; immune-competent mice	Estrogen receptor–positive breast cancer	ALDH+, Sca1/CD90 markers, label-retention, sphere assays	NK depletion, STING agonist (MSA-2)	Metastatic dormancy and late relapse	NK cells regulate CSC dormancy via STING/BACH1/SOX2 axis	Dormant CSCs evade NK cytotoxicity through decreased STING signaling and elevated BACH1/SOX2; STING activation re-sensitizes CSCs to NK killing, restoring control of metastatic dormancy.	Robust preclinical + immuno logical	Moderate (relevant immune model but in murine systems only, lacking clinical correlation)
Chen et al (2023) [13]	In vitro + in vivo + trans criptomics	Human HCC TICs (CD133+); PDX mice; drug screening; RNA-seq	HCC	CD133 sorting, NANOG-GFP reporter, spheroid assays	ATRA + HDAC inhibitors (SAHA, romidepsin), anti-PD-L1	Tumor recurrence and resistance	miR22HG suppression, PTEN/TET2 upregulation, NF-κB/TLR pathway inhibition	ATRA+HDACi selectively eradicates TICs, inhibits self-renewal, enhances apoptosis and immune sensitivity; miR-22 downregulation and PTEN/TET2 restoration implicated in loss of stemness; durable response seen in PDX mice, especially when combined with immune checkpoint inhibition.	Translational + PDX model	Moderate (strong mechanistic data but lacks randomized or large-scale human validation)
Croker *et al* (2009) [28]	*In vitro* + *in vivo* functional	Breast cancer cell lines (MDA-MB-231, MDA-MB-468); NOD/SCID-IL2Rγ null mice	Breast cancer	ALDH activity (ALDEFLUOR), CD44+/CD24–, CD133	None (experimental sorting only)	Metastatic potential post-injection	CSC marker expression and ALDH activity linked to metastasis	ALDHhiCD44+CD24– and ALDHhiCD44+CD133+ cells show increased tumorigenicity, migration, invasion, and lung metastasis versus ALDHlow cells; CSC-enriched populations display enhanced malignant and metastatic capacity.	Strong preclinical	Moderate (no direct therapy or recurrence tracking; CSC link based on marker expression only)
Erdem *et al* (2024) [14]	*In vitro* + PDO + PDX + trans criptomics	CRC cell lines (SW480, HCT116, SW620), patient-derived organoids and xenografts	Colorectal cancer	CD44-high, ALDH1+ sorting, LGR5, NANOG, qRT-PCR	None (MACC1 manipulation only)	CSC-driven tumor recurrence and metastasis	MACC1 transcriptionally regulates LGR5 and stemness phenotype	MACC1 expression enhances LGR5, NANOG, and stemness in CRC PDOs and PDXs; its inhibition reduces sphere formation and tumorigenicity; MACC1 acts as transcriptional activator of LGR5 and driver of stemness-related recurrence potential.	Translational multi-platform	Moderate (no therapeutic intervention tested; relies on correlation and overexpression models)
Fang *et al* (2024) [22]	*In vitro* + *in vivo* + trans criptomics	OVCAR3 and Kuramochi ovarian cancer cell lines, patient-derived CAFs, mouse xenografts	High-grade serous ovarian cancer (HGSOC)	ALDH1A1, ALDEFLUOR assay, sphere assay	Carboplatin, PORCN inhibitor (IWP2), Box5	CSC enrichment after chemo + residual tumors	Wnt5a/CREB1/BACH1 noncanonical axis in CSC maintenance	CAFs enrich OCSCs via noncanonical Wnt5a signaling; inhibition of Wnt5a or CREB1 suppresses CSC phenotype and spheroid formation; Box5 + carboplatin reduces residual CSC pool *in vivo*.	Strong translational + *in vivo*	Moderate (robust functional data but no clinical cohort for direct relapse confirmation)
Ginestier et al (2007) [17]	In vitro + in vivo + clinical IHC	Breast cancer xenografts (NOD/scid), ALDEFLUOR assay, TMA of 577 patients	Breast cancer	ALDEFLUOR assay, ALDH1 immuno staining	None (obser vational + sorting only)	Tumorigenic capacity + clinical outcome	ALDH1 as CSC marker and predictor of outcome	ALDH1+ cells display self-renewal, multipotency, tumor initiation, and poor prognosis correlation in large patient cohort; ALDH1 staining identifies CSCs in situ and is associated with reduced overall survival.	High (preclinical + clinical)	Low (includes large-scale clinical validation with independent cohorts and functional assays)
Gupta *et al* (2009) [29]	*In vitro* + *in vivo* (functional)	HMLER and HMLE breast cancer cells; 4T1 and SUM159 models; mouse xenografts	Breast cancer	CD44high/CD24low, tumorsphere assays	Salinomycin, paclitaxel (pre-treatment)	CSC-mediated tumor regrowth and metastasis	EMT-driven CSC enrichment and drug resistance; CSC-specific salinomycin toxicity	Salinomycin selectively eliminates breast CSCs *in vitro* and *in vivo*; reduces tumorsphere formation, metastatic seeding, and expression of CSC gene signatures. Paclitaxel enriches CSCs and promotes mesenchymal traits.	Strong functional + screening	Moderate (powerful mechanistic evidence but no direct validation in primary patient tumors)
Ji *et al* (2009) [30]	*In vitro* + *in vivo* + CSC sorting	MiaPaCa2 and BxPC3 pancreatic cancer cell lines; nude mice xenografts	Pancreatic cancer	CD44+/CD133+, tumorsphere assay	miR-34 mimics, lentiviral miR-34a, chemo/radiation	CSC inhibition and tumor initiation	miR-34 represses Bcl-2 and Notch to impair CSC self-renewal	miR-34 restoration suppresses pancreatic CSCs, induces apoptosis, enhances chemo/radiosensitivity, and reduces *in vivo* tumor initiation by 87%. Targets Bcl-2 and Notch pathways; provides functional replacement for p53 loss in CSC regulation.	Translational (RNA-based)	Moderate (convincing results but performed only in two p53-mutant cell lines; no clinical samples)
Kim *et al* (2011) [31]	*In vitro* + *in vivo* (functional)	Breast, liver, sarcoma cell lines (MCF7, MDA-MB231, MES-SA/Dx5); mouse models	Various solid tumors	Functional CSC inhibition (no sorting used)	Salinomycin, Doxorubicin, Etoposide	Drug resistance and therapeutic relapse	Increased DNA damage, downregulation of p21 via proteasome activation	Salinomycin sensitizes cells to DOX/ETO by increasing DNA breaks, p53 activation, and apoptosis. Co-treatment enhances therapy-induced cytotoxicity, especially in resistant cell lines; mechanistically involves reduced p21 and increased DNA repair protein phosphorylation.	Experimental pharmaco logic	Moderate (strong mechanistic data but lacks specific CSC isolation or relapse tracking)
Li et al (2024) [24]	In vitro + in vivo + omicsz	Cervical basal epithelial cells, HaCaT, SiHa, HeLa; PDX models	Cervical cancer (SIL to SCC)	CD44+/CD326+, CK17+, sphere assays	PIWIL2 overe xpression, PDK1 modulation, DDP, DCA	Progression, regression, relapse in SIL	PIWIL2→LIN28/let-7→PDK1 axis drives glycolysis, maintains TIC stemness via mTOR	PIWIL2 upregulates PDK1 via LIN28/let-7, activating glycolysis and PI3K/AKT/mTOR, maintaining TIC markers and transcriptional reprogramming. PDK1 silencing reduces tumorigenicity, stemness, and enhances response to cisplatin (DDP) in PDX models.	Strong translational + PDX	Moderate (robust multimodal data, but cervical SIL remains a preinvasive model without long-term patient tracking)
Li *et al* (2025) [21]	*In vitro* + *in vivo* + omics	GSC models (GSC3565, GSC1914); PDX mice; RNA-seq + ATAC-seq + ChIP-seq	Glioblastoma	OLIG2+, NES+, SOX2+; tumorsphere; ELDA	Chaetocin (SUV39H1 inhibitor), TMZ	GSC-driven recurrence and resistance	SUV39H1 maintains chromatin accessibility and stemness in GSCs	SUV39H1 is overexpressed in GSCs via super-enhancer activity. Its knockdown impairs cell cycle, stemness, and chromatin accessibility, sensitizes GSCs to TMZ, and reduces tumor formation *in vivo*. Identified as a therapeutic and prognostic target for GBM.	Strong mechanistic + epigenetic	Moderate (excellent mechanistic and *in vivo* evidence, but no clinical trials or longitudinal relapse tracking)
Lopez *et al* [2]	*In vitro* + *ex vivo* immuno phenotypic profiling	6 chordoma cell lines; 18 patient tumor samples; flow cytometry + multispectral IHC	Chordoma	CD24+CD133+ (*in vitro*), CD15+CD24+ ALDH+ (tumor tissue)	None (phenotypic profiling only)	CSCs as driver of recurrence	PD-L1, B7H6, ULBP1, MICA-B overexpressed on CSCs	CSCs identified *in vitro* and *ex vivo* using dual/triple markers; PD-L1 and NK ligands highly expressed on CSCs versus non-CSCs. CSCs spatially clustered in chordoma tissue. Suggests immunotherapy (anti-PD-L1, NK strategies) as potential anti-CSC approach in recurrent chordoma.	Translational observational	Moderate (unique data on CSC immuno phenotype but lacks therapeutic testing or functional relapse model)
Mediratta *et al* (2024) [32]	*In vitro* + *in vivo* + PDX organotypic	Human TNBC cell lines (MDA-MB-231, SUM149-PT), AT3ova syngeneic model, PDX cultures (HCI-001…)	TNBC	CD44high CD24low, ALDHhigh, tumorsphere assays	Quercetin, Luteolin, Paclitaxel	Chemo therapy-induced CSC enrichment	CD73/YAP/Wnt signaling; immune suppression	CD73 upregulated by paclitaxel promotes CSCs and immune evasion; co-inhibition of CD73, YAP, and Wnt with quercetin/luteolin reduces CSC populations, restores lymphocyte infiltration, and suppresses tumor growth in TNBC models and patient-derived explants.	Translational (multi-platform)	Moderate (strong evidence but no longitudinal *in vivo* relapse or clinical validation)
Peyvandi et al (2024) [33]	In vitro + in vivo + trans criptomics	Murine 4T1 and D2A1 breast cancer models; MACS isolation; Gr1+CD11b+ immune cell co-cultures	Breast cancer (murine)	SCA1+ marker, mammo sphere, metastasis assays	JAK inhibition (ruxolitinib); Gr1+CD11b+ immune priming	CSC plasticity, metastasis post-treatment	OSM/IL-6/JAK signaling drives CSC conversion and metastasis	Gr1+CD11b+ tumor-educated cells induce SCA1+ CSCs via OSM/IL-6; plasticity of non-CSCs toward metastatic CSCs is mediated by paracrine signaling; JAK inhibition blocks this effect; signature correlates with OS/RFS and lung metastasis in human breast cancer datasets.	Robust mechanistic + scRNAseq	Moderate (clear preclinical evidence, but no direct CSC elimination or patient-level validation)
Shu *et al* (2022) [34]	*In vitro* + *in vivo* + TMA	NSCLC cell lines (A549, NCI-H226, etc.), xenograft mice, clinical samples	NSCLC	ALDEFLUOR, CD44, CD133, SOX2, Nanog, OCT4	Taxol, Wnt-C59	CSC drug resistance + tumorigenicity	Palladin induces Wnt/β-catenin activation and EMT	Palladin promotes CSC-like phenotype via Wnt3a, LRP6, GSK3β, LEF1, c-Myc; knockdown reduces taxol resistance, sphere formation, tumor growth; IHC confirms association between Palladin and β-catenin in clinical NSCLC tissues.	Translational preclinical	Moderate (robust functional assays + patient samples, but no relapse follow-up or therapeutic trial)
Song *et al* (2018) [18]	*In vitro* + *in vivo* + clinical correlation	HCC cell lines (HepG2, LM3), nude mice, 197 HCC patient specimens	HCC	Sphere assay, side population (SP), stem marker qPCR	TFAP4 over expression/silencing, luciferase	TIC phenotype and relapse correlation	TFAP4 induces DVL1 and LEF1 transcription, activating Wnt/β-catenin	TFAP4 is overexpressed in relapse-prone HCC; promotes sphere formation, SP cell fraction, tumorigenesis. Directly activates DVL1 and LEF1 via promoter binding. Correlated with poor relapse-free survival in 197 patient cohort.	High (preclinical + clinical)	Low (includes large-scale patient cohort, *in vivo* tumorigenicity, and mechanistic validation)
Xue *et al* (2024) [25]	*In vitro* + *in vivo* + omics	HepG2, Huh7, Hep3B, NSG mice, METTL16 KO, HDTVi model	HCC	CD133+, EpCAM+, tumorsphere, ELDA	METTL16 KO, eIF3a/eIF3b silencing	CSC-driven tumor initiation and maintenance	METTL16 regulates ribosome biogenesis and mRNA translation via eIF3a	METTL16 is essential for liver CSC maintenance via nucleolar localization, rRNA maturation, and translation regulation; KO reduces CSC frequency and tumor growth *in vivo*. eIF3a identified as a key target. METTL16 KO spares normal hepatocytes, suggesting therapeutic selectivity.	High (multi-omics + *in vivo*)	Moderate (very strong mechanistic data, but no patient-level validation or relapse outcome tracking)
Thayer et al (2003) [35]	In vitro + transgenic + xenograft	Pdx1-Shh transgenic mice, human PanIN lesions, SMO+ pancreatic cancer cell lines	Pancreatic adeno carcinoma	Not CSC-specific; PanIN resemblance, apoptosis assays	Cyclopamine (Hh pathway inhibitor)	Precursor lesion to advanced tumor transition	Hedgehog pathway (SHH–>SMO–>GLI1) in tumorigenesis	Misexpression of SHH drives PanIN formation in transgenic mice; pathway remains active in metastatic cell lines. Cyclopamine inhibits tumor growth in responsive SMO-high cells. HH pathway proposed as an early and late mediator of pancreatic cancer progression and survival.	Foundational preclinical	Moderate (critical discovery, but lacks CSC isolation or relapse-specific markers)
Zheng *et al* (2023) [36]	*In vitro* + *in vivo* + multi-omics	PDAC PDX models (PDX215/253/354), sphere assays, CRISPR, scRNAseq, ATAC-seq	Pancreatic ductal adeno carcinoma	CD133+, sphere formation, gene signatures	Cpd3 (NR5A2 inhibitor), siRNA, SOX2 inhibition	CSC enrichment and chemo resistance	NR5A2 drives SOX2–MYC axis and maintains CSC chromatin state	NR5A2 is overexpressed in PDAC CSCs; its inhibition reduces CD133+ CSC population, impairs sphere formation, restores chemosensitivity to gemcitabine. Chromatin remodeling and SOX2 repression mediate the CSC reprogramming effect.	Very high (multi-platform, PDX)	Low (strong functional, trans criptomic, and clinical-level validation)
Sarkar *et al* (2024) [37]	*In vitro* + *in vivo* (nano-delivery)	TNBC cell lines (MDA-MB-231, 4T1); CSCs from spheres; 4T1 orthotopic mice	TNBC	CD44+/CD24– sphere-derived CSCs, EMT markers	DOX, DIM, e-DDMSNP nano formulation	EMT-driven metastasis and recurrence	DOX-induced EMT in CSCs reversed by DIM via e-DDMSNP nanocarriers	DOX induces EMT and enhances metastasis risk via CSC enrichment. Co-delivery of DIM and DOX in exosome-sheathed MSNP (e-DDMSNP) reduces EMT, CSC markers, tumor initiation and lung metastasis *in vivo*. Median survival and tumor volume improved versus DOX alone.	Solid translational nano medicine	Moderate (clear therapeutic effect on CSCs and metastasis but lacks large-scale clinical validation)
